# Podometrics as a translational platform for kidney health and disease: Oshima award address 2025

**DOI:** 10.1007/s10157-026-02846-5

**Published:** 2026-04-07

**Authors:** Kotaro Haruhara

**Affiliations:** Division of Nephrology and Hypertension, Department of Internal Medicine, The Jikei University School of Medicine, 3-25-8 Nishi-Shimbashi Minato-ku, Tokyo, 105-8641 Japan

**Keywords:** Chronic kidney disease, Kidney function, Nephron, Podocyte, Podometrics

## Abstract

Podocyte injury and loss are pivotal early events in the development and progression of chronic kidney disease (CKD). As podocytes have a limited capacity for regeneration and proliferation, quantitative changes in podocyte number, size, and density are critical determinants of kidney health and disease. Experimental studies have shown that podocyte depletion alone is sufficient to induce albuminuria and glomerulosclerosis and that increased podocyte volume occurs in response to glomerular hypertrophy and various stresses on podocytes. These findings led to the emergence of podometrics (i.e., the quantitative assessment of podocyte number, size, density, glomerular volume, and podocyte loss rates in kidney tissue and urine). Although the clinical application of podometrics has long been limited by technical challenges, recent methodological advances have enabled reliable podometric analyses of human kidney specimens. Accumulating clinical evidence indicates that podocyte depletion is closely associated with aging, hypertension, and various kidney diseases and that podometrics provides prognostic information, including short-term therapeutic responsiveness and long-term kidney outcomes. In this article, we summarize recent advances in podometric methodologies and review the clinical and morphological factors associated with podometrics across life courses and disease states. We further introduce the concept of “nephro-podometrics”, defined as the integrated quantitative assessment of podometrics and nephron-related parameters, including nephron number and single-nephron indices, within the same kidney. This framework maximizes the information obtainable from kidney specimens and provides a quantitative pathological platform that links structural changes to functional abnormalities, thereby supporting patient feedback and clinical decision making in nephrology.

## Introduction

Podocyte injury and loss are pivotal early events in the development and progression of chronic kidney disease (CKD) [1]. Podocytes are located outside the glomerular capillary and regulate the glomerular filtration function. Observations from rodent experiments suggest that podocytes have a limited capacity to regenerate or proliferate under normal conditions [2]. The number of podocytes is a critical determinant of both normal kidney function and susceptibility to kidney disease. Wharram et al. developed a transgenic rat model in which human diphtheria toxin receptor was selectively expressed in podocytes [3]. After the injection of diphtheria toxin, they found a dose-dependent relationship between podocyte depletion and glomerular injury, as evidenced by increased albuminuria and severe glomerular injury. This is the first direct evidence that podocyte injury alone is sufficient to induce glomerular injury and that the degree of podocyte depletion affects the severity of glomerular injuries. Accumulating evidence supports the podocyte depletion hypothesis that effective and progressive reduction in podocyte number is a common pathway for glomerulosclerosis and kidney failure in most glomerular diseases [4]. Since effective increases in podocyte number are limited, increasing podocyte volume is also an important process to maintain the coverage of the glomerular capillary surface. Fukuda et al. observed a quadratic increase in glomerular volume as well as an increase in total podocyte volume that was not proportional to glomerular volume, along with body weight gain in normal rats, leading to a failure of podocyte coverage on the glomerular surface under conditions of glomerular hypertrophy [5]. Furthermore, impaired increasing podocyte volume causes early kidney failure in a genetically engineered rat model of the mTORC1 pathway, specifically in podocytes [5]. These findings suggest that podocyte size plays a critical role in compensating for the mechanical and metabolic stresses imposed on the glomerulus.

Wiggins et al. coined the term “podometrics” to describe methodologies and/or quantitative estimates of podocyte number, size, density, glomerular volume and other parameters in kidney biopsies, and the rate of podocyte detachment from glomeruli into urine [6]. Although the importance of podometrics in kidney health and disease has been well-established in experimental studies, its application in clinical settings has long been limited owing to methodological challenges. Recent advances in podometric approaches have provided novel insights into kidney health and disease in humans.

**Fig. 1:**
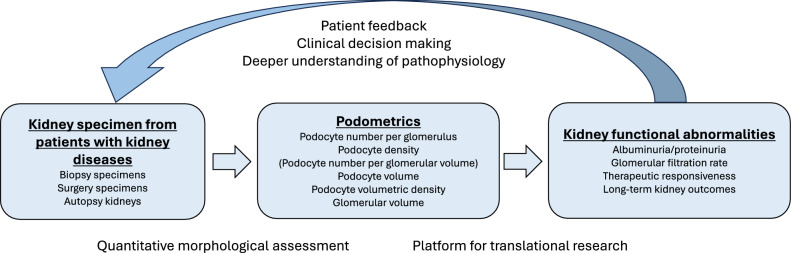
Podometrics as a translational platform linking kidney morphology to functional abnormalities. Podometrics is not the primary focus of translational research, but rather a platform that can accelerate various translational studies in nephrology. Kidney specimens obtained from biopsy, surgical nephrectomy, and autopsy are the gold standard to diagnose kidney disease under qualitative and quantitative pathological analyses. Podometrics provides quantitative information, including podocyte number per glomerulus, podocyte density (podocyte number per glomerular volume), podocyte volume, podocyte volumetric density, and glomerular volume. This quantitative morphological assessment serves as a translational platform that links kidney morphological alterations to functional abnormalities, such as albuminuria, glomerular filtration rate, therapeutic responsiveness, and long-term kidney outcomes. By integrating pathological findings with functional and clinical information, podometrics facilitates patient feedback, clinical decision making, and a deeper understanding of disease pathophysiology, thereby supporting translational research in nephrology

In this review, we summarize recent advances in podometric methodologies and the clinical and morphological factors associated with podometrics across life courses and disease states. We further introduce the novel concept of “nephron-podometrics”, defined as the simultaneous quantitative assessment of podometrics and nephron-related parameters, including nephron number and indices at the single-nephron level, within the same kidney [7]. This approach aims to maximize the information obtained from kidney specimens and understand podocyte depletion in the context of nephron mass reduction. Podometrics provides a quantitative pathological platform that links morphological changes in the kidney to functional abnormalities. This would assist physicians in providing patient feedback, making clinical decisions, and developing a deeper understanding of the pathophysiology of kidney disease (Fig. 1).

## How to measure podometrics?

Because of the spatial complexity of podocytes outside the glomerular capillary, accurately counting podocytes within a glomerulus is technically challenging. When specimens are adequately available, design-based stereology is the gold-standard method for counting the targets of interest (e.g., podocytes). Bertram et al. established a design-based stereology for counting glomerular numbers and glomerular cell types, including podocytes, in normal rat kidneys [8]. This method required multiple histological sections, and the glomerular cell types were identified by transmission electron microscopy. Puelles and Bertram et al. further developed the estimation of podocyte number using serial histological sectioning from autopsy kidneys, triple label immunohistochemistry (a podocyte marker, non-podocyte marker, and DAPI), laser confocal microscopy, and cell counting with an optical disector/fractionator [9, 10]. The major advantages of these methods based on design-based stereology are the minimization of biases due to the distribution, size, and shape of the podocytes, and the ability to obtain individual podocyte numbers and glomerular volumes in each glomerulus. In contrast, design-based stereology requires adequate kidney samples, such as autopsy kidneys, and a tremendous amount of time and effort.

In the 1970 s, Weibel and Gomez developed a model-based stereology to estimate podocyte density (podocyte number per glomerular volume) [11]. This method requires the number of podocyte nuclei per section and the ratio of podocyte volume to glomerular volume by the estimated grid point method under the assumption of a coefficient for the size distribution and shape of podocytes. Weibel and Gomez equations are frequently applied in animal and human studies [12–14]. However, a study showed the Weibel and Gomez methods overestimated podocyte number by 54% relative to estimation by design-based stereology as a gold standard in normal rodent kidneys [15].

**Fig. 2:**
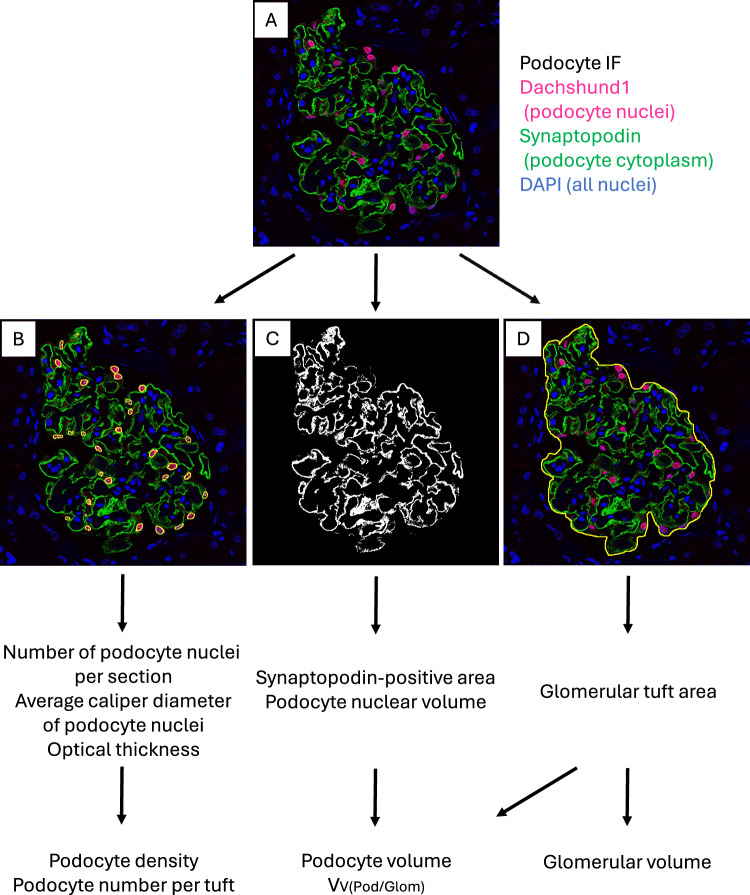
Schematic illustration of the estimation of podometrics and glomerular volume. **A** Paraffin sections were immunostained with podocyte markers using primary antibodies directed against dachshund1 (podocyte nuclei) and synaptopodin (podocyte cytoplasm). All nuclei were visualized with 4′,6-diamidino-2-phenylindole (DAPI). **B** Podocyte nuclei were identified on the podocyte immunofluorescence. Podocyte density and podocyte number per tuft were calculated based on the number of podocyte nuclei per section, average *XY*-axis caliper diameter of podocyte nuclei, total glomerular area, and optical section thickness. **C** Podocyte volume was calculated as the sum of podocyte cytoplasmic and nuclear volumes. The podocyte cytoplasm defined as being positive for SNP. The podocyte nuclear volume was estimated on the basis of the mean apparent caliper diameter of podocyte nuclei. **D** Each glomerular tuft area was measured as the area of the outer side of the capillary loops on the immunofluorescence image. The mean glomerular area for each subject was calculated by averaging measured areas of all sampled glomerular tufts. Glomerular volume was calculated from the mean glomerular area using the Weibel and Gomez equation. Reprinted from Haruhara, K., et al. "Associations between nephron number and podometrics in human kidneys." *Kidney Int* 102(5): 1127–1135 [26]. Copyright (2022), with permission from Elsevier

To overcome the above accuracy issue, Venkatareddy and Wiggins et al. introduced the correlation factor method using an equation that incorporates the known histological section thickness and measures the mean diameter of podocyte nuclei to estimate podocyte density [16]. Podocyte nuclei were identified by immunofluorescence using the TLE4. Podocyte volume was also estimated based on immunohistochemistry using the podocyte cytoplasm marker GLEPP1 [17, 18]. We have reported some modifications of the Venkatareddy method: i) immunofluorescence with dual podocyte markers dachshund 1 (podocyte nuclei marker) and synaptopodin (podocyte cytoplasm marker) and ii) imaging by confocal microscopy [19]. These modifications enabled us to identify podocyte nuclei and obtain accurate optical section thickness, which is required to estimate podocyte density (Fig. 2).

## How is podometrics associated with clinical and morphological factors?

### Low birth weight and premature birth

As podocytes have a limited capacity for regeneration or proliferation, the number of podocytes at birth (podocyte endowment) is critical in determining kidney health and disease. Ikezumi et al. reported that the number of podocytes in children with FSGS and a history of low birth weight (LBW) was 24% lower than that in those without LBW [12]. We reported that three young adult patients presenting with proteinuria and a previous history of LBW had a lower podocyte density, a lower number of podocytes per glomerulus, and a lower number of non-sclerotic glomeruli [20]. An animal study demonstrated that maternal malnutrition induced by a low-protein diet confers lower podocyte endowment in offspring rats, lower LBW, and fewer nephrons [21]. These findings suggest that LBW and premature birth are associated with lower podocyte endowment, which potentially contributes to the development of kidney disease later in life. Given the accumulating evidence suggesting that LBW and premature birth are associated with CKD [22], podocyte number may be one of the key components in mediating this association.

### Podocyte gains during childhood

In contrast to the number of nephrons, an increase in the number of podocytes may occur during childhood. An autopsy study assessed podometrics using design-based stereology in 4 children (median age, 1.75 years) and 12 adults (median age, 40 years) without overt kidney disease, revealing that children and adults had a median of 452 and 558 podocytes per glomerulus, respectively [10]. A recent study using kidneys obtained from forensic autopsy cases of Japanese children without kidney disease suggested that podocyte numbers increased up to approximately 36 months of age [23]. These findings suggest that podocyte number can increase during childhood, and understanding the regulation of this increase would provide a novel therapeutic target in subjects with lower podocyte endowment.

### Aging

Prior studies have indicated that podocyte numbers decrease with normal aging [24–27]. Hodgin et al. reported that podocyte density and number per glomerulus decreased, whereas glomerular volume increased with age in 89 kidney samples from living and deceased kidney donors and normal poles of nephrectomies [24]. A design-based stereology study in 19 adult Caucasian American males without overt kidney disease showed that older age was associated with lower podocyte density and podocyte number per glomerulus, but not with increased glomerular volume [25]. Our autopsy study in 50 Japanese subjects without apparent glomerular disease reported that total podocyte number per kidney, which was estimated by multiplying the number of non-sclerotic glomeruli per kidney by the number of podocytes per glomerulus, was inversely correlated with age, with an annual loss of approximately 5.6 million podocytes per kidney per year [27]. This age-related podocyte loss might be, at least in part, associated with increased vulnerability to the development and/or progression of CKD in the elderly population.

### Hypertension

Hypertension is another possible factor related to accelerated podocyte depletion. An autopsy study using design-based stereology showed that hypertension was associated with a larger glomerular volume and a lower podocyte density, but not with podocyte number per glomerulus [28]. In our Japanese autopsy series, hypertensive subjects had significantly fewer podocytes per tuft than normotensive subjects. Furthermore, the presence of hypertension was associated with a lower number of podocytes in juxtamedullary glomeruli, whereas older age was associated with fewer podocytes in superficial glomeruli in a two-factorial analysis of variance [26]. A study by Naik et al. showed that a higher mean blood pressure, even within the normal range, was significantly associated with an increased amount of podocyte mRNA in the urine of healthy living kidney donors [29]. These findings suggest that hypertension and elevated blood pressure contribute to podocyte depletion. Thus, optimal blood pressure control may be essential to prevent further podocyte loss and depletion.

### Kidney diseases

Podocyte number is decreased in various kidney diseases, such as diabetic nephropathy [30–32], benign nephrosclerosis [33], IgA nephropathy [13], Alport syndrome [18], and obesity-related glomerulopathy (ORG) [14, 34]. We analyzed podometrics in biopsy specimens from 46 ORG patients using our method based on model-based stereology [34]. At the time of the diagnostic biopsy, podocyte density and podocyte number per glomerulus in ORG patients with preserved kidney function (eGFR > 60 mL/min/1.73 m^2^) were lower than those in kidney donors with obesity (body mass index > 25 kg/m^2^), whereas non-sclerotic glomerular number and kidney function were comparable between them. This finding suggests that podocyte depletion is an early event and a possible predisposing factor for the development of ORG in obese individuals. Furthermore, lower podocyte density at the time of the diagnosis, but not podocyte number per glomerulus, was associated with worse kidney outcomes in 46 ORG patients, even after adjustment for clinical variables. Another podometric study reported that podocyte number per glomerulus in primary FSGS patients with a good response to first-line therapy at 6 months was greater than in those with poor response, suggesting that podocyte depletion is associated with worse therapeutic responsiveness [35]. These findings suggest that podometric assessment at the time of a diagnostic biopsy may provide additional information regarding therapeutic responsiveness and subsequent kidney outcomes. Further investigation is needed to elucidate how podometrics can predict clinical trajectories, including both short-term therapeutic responsiveness and long-term kidney outcomes, in specific glomerular diseases.

### Glomerular volume and nephron number

Podometrics is intrinsically associated with nephron-related parameters, including glomerular volume and nephron number [10, 19, 26]. One of the major advantages of design-based stereology is that it allows podometrics to be evaluated in individual glomeruli. Puelles et al. demonstrated that individual glomerular volume is indirectly correlated with podocyte density and directly correlated with podocyte number per glomerulus [10]. Increased glomerular volume, namely, glomerular hypertrophy, is accompanied by various factors, including body size growth, nephron mass reduction, obesity, and diabetes, suggesting that glomerular hypertrophy is a compensatory mechanism to reduce intra-glomerular pressure against glomerular stresses. Taken together, the podocyte number potentially regulates maximum glomerular hypertrophy. In our autopsy study, we found that the number of non-sclerotic glomeruli measured using design-based stereology was directly correlated with podometrics, including podocyte density, podocyte number per glomeruli, and podocyte volumetric density (ratio of total podocyte volume to glomerular volume) [26]. Since these podometric parameters are all indicative of kidney health, it is suggested that healthy kidneys have lost fewer nephrons and podocytes in adult life and/or have a higher nephron and podocyte endowment at the commencement of postnatal life. These findings led us to realize that it is important to consider both podometrics and nephron-related parameters when analyzing kidney specimens.

## What is the concept of nephro-podometrics?

As discussed above, podometrics is intrinsically linked to nephron-related parameters, including nephron number, glomerular volume, and indices at the single-nephron level. A reduction in nephron mass, whether due to a congenitally low number of nephrons, aging, or kidney disease, leads to increased single-nephron glomerular filtration rate and elevated intraglomerular pressure. These are the major drivers of glomerular hypertrophy and mechanical stress in the podocytes. As podocytes have a limited regenerative capacity, sustained stress can lead to podocyte detachment and/or apoptosis. This ultimately results in glomerulosclerosis and nephron loss. Thus, reduction in nephron mass and podocyte depletion potentiate each other, forming a vicious cycle that accelerates the decline in the kidney function. To better understand these relevant yet interdependent processes, simultaneous quantitative assessment of both nephron-related parameters and podometrics within the same kidney is required [19, 26, 34]. We have proposed the concept of “nephro-podometrics” as an integrated morphometric framework combining podometrics with the quantification of nephron-related parameters [7]. This approach enables evaluation indices at both the whole-body level, such as total podocyte number per kidney, and the single-nephron level, which cannot be assessed in isolation. Using this integrated framework, we demonstrated that nephro-podometrics provides functional insights when considered in the context of remnant nephron masses [19, 26, 27, 34]. For example, we found an inverse correlation between podocyte density and the single-nephron estimated glomerular filtration rate (as calculated by whole body eGFR divided by the number of non-sclerotic glomeruli) in ORG patients. This indicates that reduced podocyte density and glomerular hyperfiltration jointly contribute to the progressive decline in the kidney function [26]. Nephro-podometrics can capture pathophysiological mechanisms that are not fully explained by the conventional evaluation of glomerular hyperfiltration or podocyte depletion alone [7].

## Future perspectives

Podometrics has emerged as a quantitative approach that substantially expands the morphological information obtained from kidney specimens. The combination with state-of-the-art technology would further improve the accuracy and reliability of podometrics. The whole-glomerular scan technique using the tissue optical clearing method contributes to accurate podometrics in individual glomeruli [21, 28, 36]. This method requires kidney specimens of sufficient size, which makes it difficult to apply to clinical biopsy specimens. However, a recent study utilized tissue optical clearing methods to detect crescentic lesions within the entire glomerulus in human kidney biopsy specimens [37]. Another breakthrough is the application of artificial intelligence, including deep-learning algorithms. Current podometrics methods mostly require labor-intensive manual annotations for podocytes for immunostaining podocyte markers. Some researchers have successfully developed deep-learning algorithms to detect and segment podocytes [38, 39]. Digital pathology and computational image analysis, combined with deep-learning technology, are research areas that have recently emerged as an alternative to the conventional qualitative or semi-quantitative assessment of pathology [40, 41]. These technical advances will further improve podometrics as part of pathomics, in which quantitative features are systematically extracted from histopathological images to capture clinically important structural information. Taken together, the improved accuracy and reliability of podometrics using these novel technologies would contribute to maximizing information from clinical kidney tissues and provide valuable information and insights for nephrologists and patients with kidney diseases.

In conclusion, podometrics, as well as estimating nephron-related parameters, provide quantitative and clinically informative platforms that potentially link pathophysiological assumptions regarding morphological changes and functional abnormalities in clinical kidney specimens. Further improvement in podometrics and expansion of its clinical application would be beneficial for nephrologists and pathologists in advancing patient care for kidney disease.

## Data Availability

This article is not applicable.
